# Lifetime Blast Exposure Is Not Related to White Matter Integrity in Service Members and Veterans With and Without Uncomplicated Mild Traumatic Brain Injury

**DOI:** 10.1089/neur.2023.0043

**Published:** 2023-12-14

**Authors:** Sara M. Lippa, Ping-Hong Yeh, Jan E. Kennedy, Jason M. Bailie, John Ollinger, Tracey A. Brickell, Louis M. French, Rael T. Lange

**Affiliations:** ^1^ Walter Reed National Military Medical Center, Bethesda, Maryland, USA.; ^2^ National Intrepid Center of Excellence, Bethesda, Maryland, USA.; ^3^ Uniformed Services University of the Health Sciences, Bethesda, Maryland, USA.; ^4^ Traumatic Brain Injury Center of Excellence, Silver Spring, Maryland, USA.; ^5^ Contractor, General Dynamics Information Technology, Silver Spring, Maryland, USA.; ^6^ Brooke Army Medical Center, Joint Base, San Antonio, Texas, USA.; ^7^ 33 Area Branch Clinic, Camp Pendleton, California, USA.; ^8^ University of British Columbia, Vancouver, British Columbia, USA.

**Keywords:** blast, diffusion tensor imaging, military, subconcussive, traumatic brain injury

## Abstract

This study examines the impact of lifetime blast exposure on white matter integrity in service members and veterans (SMVs). Participants were 227 SMVs, including those with a history of mild traumatic brain injury (mTBI; *n* = 124), orthopedic injury controls (*n* = 58), and non-injured controls (*n* = 45), prospectively enrolled in a Defense and Veterans Brain Injury Center (DVBIC)/Traumatic Brain Injury Center of Excellence (TBICoE) study. Participants were divided into three groups based on number of self-reported lifetime blast exposures: none (*n* = 53); low (i.e., 1–9 blasts; *n* = 81); and high (i.e., ≥10 blasts; *n* = 93). All participants underwent diffusion tensor imaging (DTI) at least 11 months post-injury. Tract-of-interest (TOI) analysis was applied to investigate fractional anisotropy and mean, radial, and axial diffusivity (AD) in left and right total cerebral white matter as well as 24 tracts. Benjamini-Hochberg false discovery rate (FDR) correction was used. Regressions investigating blast exposure and mTBI on white matter integrity, controlling for age, revealed that the presence of mTBI history was associated with lower AD in the bilateral superior longitudinal fasciculus and arcuate fasciculus and left cingulum (βs = −0.255 to −0.174; *p*s < 0.01); however, when non-injured controls were removed from the sample (but orthopedic injury controls remained), these relationships were attenuated and did not survive FDR correction. Regression models were rerun with modified post-traumatic stress disorder (PTSD) diagnosis added as a predictor. After FDR correction, PTSD was not significantly associated with white matter integrity in any of the models. Overall, there was no relationship between white matter integrity and self-reported lifetime blast exposure or PTSD.

## Introduction

Military personnel are often exposed to blasts during training and combat deployments. Indeed, blasts are one of the main causes of traumatic brain injury (TBI) in active duty service members^1^ and are also related to increased risk of post-traumatic stress disorder (PTSD).^2,3^ Though brain injury from primary blast injury alone is rare,^4^ many have questioned whether subconcussive blast exposure may negatively impact the brain, perhaps increasing vulnerability for cognitive decline^5^ and/or development of PTSD,^6^ with a call to examine the relationship between subconcussive blast exposures and pathophysiological changes as detected by advanced neuroimaging.^5^

Diffusion tensor imaging (DTI) is one such method that has often been used to assess brain damage and, specifically, changes in axonal integrity.^7–10^ Segmentation of white matter bundles using diffusion magnetic resonance imaging (MRI) fiber tractography has been identified as the best currently available method for examining white matter pathways *in vivo* in humans.^11^ DTI produces four metrics.^12^ 1) Fractional anisotropy (FA) is a summary measure of microstructural integrity, representing diffusivity out of alignment with the principal diffusion direction. 2) Axial diffusivity (AD) represents diffusivity parallel to principal diffusion direction. AD tends to decrease with axonal injury. 3) Radial diffusivity (RD) represents diffusivity perpendicular to principal diffusion direction, which usually increases with demyelination. 4) Mean diffusivity (MD) is an indicator of overall diffusivity and is an inverse measure of membrane density. Although DTI is sensitive to white matter changes, the relationship between DTI metrics and underlying pathological changes is complex and difficult to characterize. Therefore, it is recommended that interpretations be made considering all DTI metrics.^13^

Proposed mechanisms for how blast exposure might reduce white matter integrity include disrupted blood flow to the brain^15^ or traumatic axonal injury through acceleration/deceleration forces. Blast shock wave propagation can result in mechanical damage, particularly to interfaces between structures of different densities and elasticity, leading to axonal stretch and disruption.^17^ These findings may support the postulate that blast waves ripple through the torso up into the brain through the major great vessels,^18,19^ leading to shear-strain deformation and resulting in multi-focal scattered lesions commonly observed in cerebral gray/white matter junctions, the deep subcortical white matter tracts, centrum semiovale, the dorsolateral aspect of the upper brain stem, basal ganglion, and cerebellum.^4,20^

Importantly, blast exposure is difficult to estimate, particularly across one's lifetime. Whereas pressure sensors have been used to assess blast exposure over relatively short intervals during training,^21,22^ lifetime blast exposure has generally been conducted by assessment methods varying from a single question^6,23–26^ to structured interviews assessing myriad types of weapon systems (for a review, see Turner and colleagues)^27^ and interviews that account for distance from the blast.^28,29^ No measure is able to accurately capture the force of individual blasts over a lifetime, each of which is dependent on environmental factors including distance from the original blast, whether the blast occurred in an enclosed space, personal protective equipment, and whether there were objects between a person and the source of the blast. Notably, distance from blast may be important to consider, given that there is evidence of altered functional neuroimaging in veterans with a history of close blast exposure (i.e., within 10 m), independent of concussion history.^30,31^

Multiple studies have used DTI to investigate how blast exposure may impact white matter integrity, with somewhat mixed results.^6,14–16^ Breachers have been a particularly interesting subset of persons to study, given that they are generally exposed to myriad blasts through training exercises and regular duties. Stone and colleagues compared 20 breachers (13 with a history of concussion) to 14 military/police controls (6 with a history of concussion).^14^ They found that the breachers had decreased FA and RD in a number of areas, particularly the frontal lobe white matter, corpus callosum, corticospinal tract (CST), and claustrum. In contrast, in an innovative study comparing DTI metrics before and after breacher training in 21 SWAT personnel (with no information on TBI history provided), there were no changes in FA, MD, AD, or RD.^15^ It is unclear whether history of concussion may increase one's vulnerability to subconcussive blast exposure and subsequent poor outcome.

Other studies have focused on veterans. In one investigation of the FA of 40 veterans with a history of blast exposure, number of blast exposures was related to decreased FA overall as well as in several tracts, including the splenium, bilateral CST, cingulum, right retrolenticular internal capsule and posterior corona radiata, and left sagittal stratum, fornix stria terminalis, and uncinate fasciculus.^16^ Of note, however, no correction for multiple comparisons was applied.

Bazarian and colleagues investigated FA and MD in 52 veterans.^6^ They found that those with any blast exposure had lower 1st (but not 50th or 99th) percentile FA values than those with no blast exposure. Additionally, PTSD severity was associated with higher 1st percentile MD values. When investigating whether blast exposure or PTSD was associated with FA and MD of individual tracts, however, no relationships survived correction for multiple comparisons.

In sum, previous investigations in this area have largely consisted of small sample sizes and have produced conflicting results. The present study aimed to examine how lifetime blast exposure relates to DTI metrics, obtained through diffusion MRI tractography, in service members and veterans (SMVs) with and without uncomplicated mild TBI (mTBI). Further, because exposure to and effects of blasts may have important implications for PTSD, we also aimed to explore whether there was a relationship between PTSD and white matter integrity in models that also accounted for blast exposure and mTBI history.

## Methods

### Participants

Participants were 227 U.S. SMVs (mean age = 39.6 years, SD = 10.2) prospectively enrolled in the Defense and Veterans Brain Injury Center (DVBIC)/Traumatic Brain Injury Center of Excellence (TBICoE) 15-Year Longitudinal TBI Study. Recruitment occurred through four military medical treatment facilities, including in- and outpatient treatment programs, as well as community events and intranet advertisements.

Inclusion criteria for the overall study included active-duty service members or other Defense Enrollment Eligibility Reporting System-eligible (i.e., eligible to receive military benefits) veterans; 18 years of age or older; and ability to read and understand English. Exclusion criteria of the overall study included: history of significant neurological or psychiatric condition[s] unrelated to the injury event or deployment. Additional exclusion for this study included a history of complicated mild, moderate, severe, or penetrating TBI (i.e., Glasgow Coma Scale [GCS] <13, post-traumatic amnesia [PTA] >24 h, loss of consciousness [LOC] >30 min, and/or abnormality on computed tomography [CT] or structural MRI). This research was conducted in accordance with the Declaration of Helsinki guidelines and approved by the Walter Reed National Military Medical Center Institutional Review Board. Written informed consent was obtained from all participants.

Participants were classified into three groups based on number of self-reported lifetime blast exposures: no blast exposure (*n* = 53); low blast exposure (i.e., 1–9 blasts; *n* = 81); or high blast exposure (i.e., ≥10 blasts; *n* = 93), consistent with our previous methods.^32^ All participants underwent DTI at least 11 months post-injury.

### Injury evaluation and classification

Diagnosis and classification of TBI has been described in detail previously.^33–36^ Briefly, a comprehensive lifetime TBI history, including the Ohio State University TBI identification method and an extended semistructured clinical interview, was conducted along with medical record review. Uncomplicated mTBI (n = 124) was defined as: 1) GCS = 13–15, PTA <24 h, LOC <30 min, and/or alteration of consciousness present and 2) no trauma-related intracranial abnormality on CT or structural MRI. Of the 124 persons with a history of mTBI, 72 (58%) of these persons had a history of mTBI that involved a blast exposure.

Participants were considered injured controls (*n* = 58) if they experienced an orthopedic/soft tissue injury on or after October 1, 2001 and had no history of TBI. Participants were considered non-injured controls (*n* = 45) if they did not experience an orthopedic/soft tissue injury on or after October 1, 2001 and had no history of TBI.

### Measures

Lifetime blast exposure was assessed with a single interview question: “In your life, how many times have you been close enough to an explosion in which you felt the blast wave?”

PTSD symptoms were assessed with the PTSD Checklist-Civilian version (PCL-C),^37^ a 17-item measure designed to assess Diagnostic and Statistical Manual of Mental Disorders, Fourth Edition, Text Revision (DSM-IV-TR)^38^ symptom criteria for PTSD. Total score was used to represent total PTSD symptom burden, and individual item responses were mapped onto Diagnostic and Statistical Manual of Mental Disorders, Fourth Edition (DSM-IV)^38^ criteria B, C, and D for PTSD. Persons who met criteria B, C, and D for PTSD based on moderate or greater endorsement of the requisite items were considered to have met modified DSM-IV criteria for PTSD.

The Minnesota Multiphasic Personality Inventory, 2nd Edition, Restructured Format (MMPI-2-RF)^39^ was used to identify invalid responding (i.e., Variable Response Inconsistency [VRIN] ≥80 T, True Response Inconsistency [TRIN] ≥80 T, Infrequent Responses [F-r] = 120 T, Infrequent Psychopathology Responses [Fp-r] ≥100 T, Cannot Say [CNS] >14) and exclude such data (*n* = 2) from analyses involving PTSD symptoms/diagnosis.

#### Neuroimaging

Participants underwent MRI at the Walter Reed National Military Medical Center (WRNMMC)^40,41^ on a 3-T scanner. Diffusion weighted imaging (DWI) data pre-processing involved correction of echo planar imaging geometric distortion using a B0 field map^42^ and correction of motion and eddy current artifacts and digital brain extraction (skull stripping), using software from the FSL toolkits.^43^ We applied the robust estimation of tensors by outlier rejection (RESTORE)^44^ method to estimate the tensor. It uses weighted least squares with a non-negativity constraint to detect and exclude possible outliers before tensor estimation. This improves tensor estimation on a voxel-by-voxel basis in the presence of physiological noise artifacts,^45^ which reduces the need for manual/subjective identification of corrupted DWI images and is especially valuable when using data with frequent motion corruption.^46^

DTI scalar images (i.e., FA, AD, MD, and RD) were computed from the tensors computed with the RESTORE method.^44^ Total cerebral white matter (CWM) was segmented based on the structural T1-weighted (T1W) image using Freesurfer (V5.3). Bilateral CWM was transformed to diffusion-weighted MRI by applying the rigid transformation matrix coregistering T1W and FA image (using FSL flirt). Mean values of DTI metrics in left and right cerebral white matter were then calculated. To implement bundle-specific tractography for tract-of-interest (TOI) analysis, an automated method using a convolutional neural network-based approach, TractSeg segmentation pipeline,^47,48^ was applied to reconstruct 24 TOIs, based on a literature review of those possibly impacted by mTBI/blast^49^: forceps minor (CC1),^50^ forceps major (CC7),^6,14,16,50,51^ and genu (CC2)^14,52^ of the corpus callosum, as well as overall corpus callosum, and superior longitudinal fasciculus (SLF),^40,50,51^ which was represented by the arcuate fasciculus and SLF-III because they comprise most of the SLF, inferior longitudinal fasciculus (ILF),^50,51^ uncinate fasciculus,^16,40^ fornix,^6^ cingulum,^16,40^ anterior thalamic radiation (ATR),^40,50–52^ inferior fronto-occipito fasciculus (IFOF),^50,51^ superior cerebellar peduncles (SCPs),^6^ and CST.^14,16,50–52^

Tractometry^53^ was implemented by resampling streamlines, assigning and averaging the DTI metrics for each centroid segment for all assigned streamline segments within the diffusion native space. Finally, the mean DTI metrics of each segmented white matter tract were calculated for further group comparisons by averaging and weighting the voxels within the “core” more heavily than those voxels at the outer extremity of the bundle that are only traversed by a small fraction of the streamlines in the pathway.

### Statistical analyses

The three blast exposure groups (no, low, and high blast exposure) were first compared in terms of demographics and PTSD symptoms through analyses of variance and chi-square tests. Effect sizes were calculated with Cohen's *d* and Cohen's *H*. A series of linear regressions were conducted to examine the impact of blast exposure (no [reference group], low, or high blast exposure) and mTBI history (TBI negative or mTBI) on each individual DTI metric, controlling for age (age is consistently correlated with white matter integrity in the literature and was strongly associated with years of military service, ρ = 0.904) and repeated combining of the low and high blast exposure groups into a single any blast exposure group. Within each DTI parameter (i.e., FA, AD, MD, and RD), the Benjamini-Hochberg false discovery rate (FDR) method was used to keep the FDR at 0.05 (e.g., correcting for 26 comparisons including 24 tracts as well as left and right total cerebral white matter). Regressions that revealed a significant impact of mTBI on DTI metrics and survived correction for multiple comparisons were repeated on a reduced data set computed by removing the non-injured control group. Finally, an exploratory set of regressions was conducted to investigate the impact of PTSD on white matter integrity by adding a modified DSM-IV PTSD diagnosis into the regression models.

The FDR was again used to correct for 26 comparisons within each DTI parameter. For all regression models, plots of residuals by the predicted values were used to assess linearity and homoscedasticity. QQ plots were used to evaluate normality assumption. The variance inflation factors of each variable in the models were used to assess multi-collinearity (all were <2).

## Results

### Blast group differences in demographics

In terms of demographic differences, the no, low, and high blast exposure groups were similar in terms of age, education, and active duty versus veteran status (*p*s > 0.05). Participants in the no blast exposure group were more likely to be women (*p*s < 0.006, *H* = 0.49–0.71), less likely to have a history of mTBI (*p*s < 0.003, *H* = 0.57–0.73), reported lower PTSD symptoms (*p*s < 0.001, *d* = 0.82–0.97), and were less likely to meet modified DSM-IV criteria for PTSD (*p*s < 0.003, *H* = 0.60–0.64) than those with low or high blast exposure. Participants in the high blast exposure group served more years in the military (*p* = 0.009, *d* = 0.94) and were more likely to be white than those in the no blast exposure group (*p* = 0.002, *H* = 0.52), and more likely to be officers than the no or low blast exposure groups (*p*s < 0.001, *H* = 0.64–0.77). There were also differences by service branch, with participants in the low blast exposure group more likely to be in the Army and less likely to be in the Navy than the no and high blast exposure groups. There were no Marines in the no blast exposure group. Members in the Air Force were more likely to be in the high blast exposure group than the low blast exposure group. See Table 1.

**Table 1.** Demographics and Clinical Characteristics by Blast Exposure Group

	***No blast exposure (n = 53)***		***Low blast exposure (n = 81)***		***High blast exposure (n = 93)***		
	***M***	***SD***	***M***	***SD***	***M***	***SD***	*p* ***values***
Age	37.0	12.7	39.5	10.2	41.2	8.2	0.077^e^
Education (years)	15.8	2.6	15.6	2.6	15.0	2.0	0.096^e^
Years of service^a^	11.4	9.3	16.1	9.1	19.4	8.0	<0.001
PCL-C total score^b^	23.7	9.8	35.4	17.3	35.7	14.0	<0.001
No. of blasts reported	0.0	0.0	3.2	2.3	352.7	608.9	<0.001

	***Median***	***IQR***	***Median***	***IQR***	***Median***	***IQR***	
No. of blasts reported	0	0–0	3	1–5	100	28–500	<0.001

	**n**	***%***	**n**	***%***	**n**	***%***	**p *values***
Male	41	77.4	76	93.8	91	97.8	<0.001
White, non-Hispanic	33	62.3	60	74.1	79	84.9	0.008
Active duty (vs. veteran)^c^	42	79.2	53	68.8	62	68.9	0.346
Officer (vs. enlisted)^d^	27	51.9	36	44.4	15	16.3	<0.001
Branch							<0.001
Air Force	6	11.3	3	3.7	12	12.9	0.095
Army	24	45.3	57	70.4	44	47.3	0.003
Marine Corps	0	0.0	10	12.3	11	11.8	0.029
Navy	21	39.6	9	11.1	26	28.0	0.001
Other	2	3.8	2	2.5	0	0.0	0.208
mTBI present	16	30.2	47	58.0	61	65.6	<0.001
PTSD present^b^	2	3.8	20	25.0	21	22.8	0.005

^a^ *n* = 48 in the no blast exposure group, *n* = 71 in the low blast exposure group, and *n* = 82 in the high blast exposure group.

^b^ *n* = 80 in the low blast exposure group and *n* = 92 in the high blast exposure group.

^c^ *n* = 77 in the low blast exposure group and *n* = 90 in the high blast exposure group.

^d^ *n* = 52 in the no blast exposure group and *n* = 92 in the high blast exposure group.

^e^ Welch's *t*-test.

mTBI, uncomplicated mild traumatic brain injury; PCL, Post-traumatic Stress Disorder Checklist; PTSD, post-traumatic stress disorder.

### The impact of blast exposure and mild traumatic brain injury on white matter integrity

A series of multi-variate linear regressions was run to investigate the impact of blast exposure and mTBI on white matter integrity, controlling for age. Blast exposure group findings did not survive FDR correction; however, before correction for multiple comparisons, low blast exposure was associated with decreased AD in the CC-2, bilateral cingulum, left uncinate fasciculus, right overall cerebral white matter, right SLF-III, arcuate fasciculus, and fornix compared to the no blast exposure group (βs = −0.225 to −0.169, *p*s < 0.05). Before correction for multiple comparisons, low blast exposure was also associated with decreased left CST RD and decreased MD in the bilateral SLF-III and arcuate fasciculus, left CST, and right fornix, compared to the no blast exposure group (βs = −0.200 to −0.162, *p*s < 0.05). FA was increased in the left IFO in the low blast exposure group compared to the no blast exposure group (β = 0.177, *p* = 0.042), but this also did not survive correction for multiple comparisons. See Table 2.

**Table 2.** Regression Coefficients for Main Models Examining How Blast Exposure and mTBI Associate With White Matter Integrity

		***AD***			***RD***			***MD***			***FA***		
		***SE***	***β***	*p* ***values***	***SE***	***β***	*p* ***values***	***SE***	***β***	*p* ***values***	***SE***	***β***	*p* ***values***
CC	LBE	6.5E-06	–0.14	0.106	8.0E-06	0.03	0.687	7.1E-06	–0.02	0.845	0.0045	–0.05	0.559
	HBE	6.5E-06	–0.14	0.116	8.0E-06	0.00	0.961	7.1E-06	–0.04	0.603	0.0045	–0.03	0.760
	mTBI	5.0E-06	0.04	0.597	6.1E-06	0.09	0.192	5.4E-06	0.08	0.253	0.0034	–0.05	0.441
CC1	LBE	9.4E-06	–0.11	0.216	9.7E-06	0.00	0.968	8.8E-06	–0.03	0.679	0.0073	0.03	0.730
	HBE	9.4E-06	–0.08	0.343	9.7E-06	–0.01	0.896	8.8E-06	–0.04	0.663	0.0073	0.05	0.546
	mTBI	7.2E-06	–0.03	0.666	7.4E-06	0.04	0.569	6.7E-06	0.02	0.791	0.0056	–0.01	0.899
CC2	LBE	6.8E-06	–0.17	0.041	8.5E-06	–0.05	0.572	7.4E-06	–0.09	0.289	0.0053	0.03	0.699
	HBE	6.8E-06	–0.15	0.098	8.5E-06	–0.05	0.585	7.4E-06	–0.08	0.354	0.0053	0.03	0.734
	mTBI	5.2E-06	–0.02	0.748	6.5E-06	0.04	0.497	5.6E-06	0.03	0.673	0.0040	–0.02	0.735
CC7	LBE	1.9E-05	0.03	0.699	2.0E-05	0.06	0.516	1.9E-05	0.05	0.560	0.0081	0.00	0.963
	HBE	1.9E-05	0.04	0.625	2.0E-05	0.06	0.507	1.9E-05	0.06	0.531	0.0081	–0.01	0.901
	mTBI	1.5E-05	0.02	0.774	1.5E-05	0.06	0.381	1.5E-05	0.05	0.479	0.0062	–0.05	0.471
CWM L	LBE	4.6E-06	–0.12	0.154	5.0E-06	–0.07	0.403	4.7E-06	–0.09	0.283	0.0027	0.01	0.886
	HBE	4.6E-06	–0.24	0.008	5.0E-06	–0.13	0.127	4.7E-06	–0.17	0.048	0.0027	–0.02	0.796
	mTBI	3.6E-06	–0.03	0.657	3.8E-06	0.01	0.927	3.6E-06	–0.01	0.935	0.0021	0.00	0.963
SLF-III L	LBE	6.3E-06	–0.13	0.109	6.2E-06	–0.16	0.051	5.7E-06	–0.17	0.043	0.0045	0.12	0.184
	HBE	6.3E-06	–0.09	0.306	6.2E-06	–0.13	0.134	5.7E-06	–0.13	0.139	0.0045	0.10	0.258
	mTBI	**4.8E-06**	**–0.20**	**0.003**	4.8E-06	–0.07	0.282	4.3E-06	–0.13	0.056	0.0035	–0.01	0.833
AF L	LBE	5.8E-06	–0.15	0.069	4.6E-06	–0.16	0.054	4.3E-06	–0.18	0.029	0.0039	0.07	0.393
	HBE	5.8E-06	–0.15	0.077	4.6E-06	–0.19	0.029	4.3E-06	–0.20	0.019	0.0039	0.09	0.335
	mTBI	**4.4E-06**	**–0.21**	**0.002**	3.5E-06	–0.07	0.319	3.3E-06	–0.14	0.033	0.0030	–0.03	0.669
ATR L	LBE	9.0E-06	–0.03	0.755	9.6E-06	–0.03	0.690	9.1E-06	–0.03	0.701	0.0043	0.10	0.225
	HBE	9.0E-06	–0.07	0.425	9.5E-06	–0.03	0.747	9.0E-06	–0.04	0.623	0.0042	0.04	0.646
	mTBI	6.9E-06	–0.03	0.595	7.3E-06	0.02	0.733	6.9E-06	0.00	0.949	0.0033	–0.05	0.460
CG L	LBE	7.4E-06	–0.22	0.009	8.0E-06	–0.07	0.402	7.2E-06	–0.13	0.126	0.0050	–0.05	0.580
	HBE	7.4E-06	–0.14	0.096	8.0E-06	0.00	0.958	7.1E-06	–0.05	0.540	0.0050	–0.08	0.373
	mTBI	**5.6E-06**	**–0.24**	**0.000**	6.1E-06	–0.08	0.216	5.5E-06	–0.15	0.029	0.0039	–0.03	0.623
CST L	LBE	7.0E-06	–0.15	0.094	6.5E-06	–0.19	0.031	5.8E-06	–0.20	0.022	0.0059	0.15	0.092
	HBE	6.9E-06	–0.06	0.468	6.5E-06	–0.22	0.014	5.8E-06	–0.19	0.033	0.0059	0.21	0.018
	mTBI	5.3E-06	–0.05	0.508	5.0E-06	–0.01	0.899	4.4E-06	–0.02	0.717	0.0045	0.03	0.704
FX L	LBE	4.5E-05	–0.09	0.297	4.2E-05	–0.06	0.451	4.3E-05	–0.07	0.388	0.0077	0.04	0.641
	HBE	4.5E-05	–0.19	0.029	4.2E-05	–0.15	0.068	4.3E-05	–0.16	0.049	0.0077	0.09	0.283
	mTBI	3.4E-05	0.13	0.057	3.2E-05	0.15	0.019	3.3E-05	0.14	0.027	0.0059	–0.15	0.017
IFO L	LBE	8.9E-06	0.06	0.512	7.8E-06	–0.04	0.614	7.7E-06	–0.01	0.932	0.0050	0.18	0.042
	HBE	8.9E-06	0.03	0.775	7.7E-06	–0.07	0.444	7.6E-06	–0.04	0.685	0.0050	0.16	0.069
	mTBI	6.8E-06	–0.17	0.016	5.9E-06	–0.06	0.389	5.9E-06	–0.10	0.127	0.0038	–0.02	0.824
ILF L	LBE	1.2E-05	0.06	0.504	9.4E-06	–0.03	0.684	9.5E-06	0.00	0.996	0.0058	0.15	0.077
	HBE	1.2E-05	0.09	0.318	9.4E-06	–0.05	0.590	9.5E-06	0.00	0.957	0.0057	0.19	0.034
	mTBI	9.0E-06	–0.15	0.033	7.2E-06	–0.04	0.599	7.3E-06	–0.08	0.221	0.0044	–0.04	0.518
SCP L	LBE	9.7E-06	–0.04	0.669	8.1E-06	–0.02	0.826	8.1E-06	–0.03	0.752	0.0048	0.08	0.360
	HBE	9.6E-06	–0.07	0.411	8.1E-06	–0.12	0.173	8.1E-06	–0.11	0.218	0.0048	0.18	0.040
	mTBI	7.4E-06	–0.02	0.792	6.2E-06	–0.05	0.512	6.2E-06	–0.04	0.589	0.0037	0.07	0.292
UF L	LBE	6.0E-06	–0.18	0.037	6.6E-06	–0.12	0.152	5.8E-06	–0.16	0.072	0.0052	0.09	0.300
	HBE	6.0E-06	–0.21	0.018	6.6E-06	–0.08	0.364	5.8E-06	–0.13	0.134	0.0052	0.02	0.787
	mTBI	4.6E-06	–0.05	0.443	5.1E-06	–0.02	0.767	4.5E-06	–0.03	0.626	0.0040	0.03	0.702
CWM R	LBE	4.6E-06	–0.22	0.009	5.0E-06	–0.07	0.435	4.6E-06	–0.12	0.152	0.0028	–0.08	0.310
	HBE	4.6E-06	–0.23	0.009	5.0E-06	–0.09	0.301	4.6E-06	–0.14	0.107	0.0028	–0.08	0.336
	mTBI	3.5E-06	–0.05	0.453	3.8E-06	–0.01	0.924	3.5E-06	–0.02	0.751	0.0021	0.00	0.983
SLF-III R	LBE	6.6E-06	–-0.22	0.007	6.3E-06	–0.14	0.103	5.8E-06	–0.18	0.028	0.0054	0.04	0.678
	HBE	6.6E-06	–-0.12	0.146	6.3E-06	–0.07	0.396	5.8E-06	–0.10	0.244	0.0054	0.03	0.768
	mTBI	**5.1E-06**	**–-0.17**	**0.009**	4.8E-06	–0.06	0.404	4.4E-06	–0.11	0.108	0.0041	0.01	0.869
AF R	LBE	5.9E-06	–-0.20	0.015	4.9E-06	–0.12	0.165	4.7E-06	–0.17	0.046	0.0041	0.01	0.890
	HBE	5.9E-06	–-0.15	0.070	4.9E-06	–0.10	0.242	4.7E-06	–0.14	0.114	0.0041	0.01	0.905
	mTBI	**4.5E-06**	**–0.25**	**0.000**	3.7E-06	–0.08	0.259	3.6E-06	–0.16	0.016	0.0031	–0.05	0.467
ATR R	LBE	8.8E-06	–0.14	0.099	9.4E-06	–0.12	0.131	8.9E-06	–0.13	0.107	0.0042	0.10	0.227
	HBE	8.8E-06	–0.18	0.034	9.4E-06	–0.11	0.176	8.9E-06	–0.14	0.098	0.0042	0.03	0.736
	mTBI	6.8E-06	0.03	0.642	7.2E-06	0.03	0.681	6.8E-06	0.03	0.657	0.0032	0.03	0.613
CG R	LBE	6.8E-06	–0.22	0.010	7.8E-06	–0.06	0.504	6.8E-06	–0.12	0.170	0.0052	–0.05	0.550
	HBE	6.8E-06	–0.08	0.342	7.8E-06	0.01	0.951	6.8E-06	–0.02	0.786	0.0051	–0.03	0.702
	mTBI	5.2E-06	–0.17	0.012	6.0E-06	–0.05	0.461	5.2E-06	–0.09	0.159	0.0039	–0.03	0.708
CST R	LBE	7.9E-06	–0.12	0.172	6.8E-06	–0.12	0.169	6.4E-06	–0.13	0.127	0.0056	0.10	0.260
	HBE	7.9E-06	–0.07	0.460	6.8E-06	–0.17	0.057	6.4E-06	–0.15	0.099	0.0056	0.16	0.076
	mTBI	6.0E-06	–0.08	0.256	5.2E-06	–0.05	0.483	4.9E-06	–0.07	0.338	0.0043	0.06	0.382
FX R	LBE	4.5E-05	–0.17	0.043	4.3E-05	–0.16	0.054	4.3E-05	–0.16	0.048	0.0074	0.14	0.085
	HBE	4.5E-05	–0.15	0.081	4.3E-05	–0.12	0.143	4.3E-05	–0.13	0.115	0.0074	0.09	0.300
	mTBI	3.5E-05	0.09	0.184	3.3E-05	0.12	0.071	3.3E-05	0.11	0.097	0.0057	–0.13	0.048
IFO R	LBE	8.2E-06	0.00	0.980	7.5E-06	0.01	0.950	7.2E-06	0.00	0.958	0.0048	0.09	0.305
	HBE	8.2E-06	0.01	0.936	7.5E-06	–0.06	0.530	7.2E-06	–0.04	0.688	0.0048	0.14	0.107
	mTBI	6.3E-06	–0.13	0.060	5.7E-06	–0.02	0.812	5.5E-06	–0.06	0.381	0.0037	–0.01	0.854
ILF R	LBE	1.2E-05	0.06	0.499	8.8E-06	0.02	0.836	9.1E-06	0.04	0.668	0.0057	0.11	0.189
	HBE	1.2E-05	0.16	0.077	8.8E-06	0.05	0.558	9.1E-06	0.10	0.249	0.0057	0.15	0.086
	mTBI	9.1E-06	–0.14	0.046	6.8E-06	–0.06	0.362	7.0E-06	–0.10	0.145	0.0044	0.00	0.981
SCP R	LBE	8.8E-06	–0.08	0.350	7.0E-06	–0.01	0.944	7.0E-06	–0.04	0.660	0.0046	0.01	0.881
	HBE	8.8E-06	–0.13	0.156	6.9E-06	–0.17	0.052	7.0E-06	–0.17	0.060	0.0046	0.16	0.059
	mTBI	6.7E-06	–0.03	0.705	5.3E-06	–0.07	0.283	5.4E-06	–0.06	0.385	0.0035	0.10	0.141
UF R	LBE	6.0E-06	–0.13	0.116	6.0E-06	–0.04	0.646	5.4E-06	–0.08	0.357	0.0049	0.04	0.611
	HBE	6.0E-06	–0.20	0.023	6.0E-06	–0.06	0.499	5.4E-06	–0.12	0.179	0.0049	0.02	0.866
	mTBI	4.6E-06	–0.12	0.089	4.6E-06	–0.03	0.690	4.2E-06	–0.06	0.355	0.0037	–0.01	0.916

Bolded results survived Benjamini-Hochberg false discovery rate correction for multiple comparisons.

AD, axial diffusivity; AF, arcuate fasciculus; ATR, anterior thalamic radiation; CC, corpus callosum; CC1, forceps minor; CC2, genu; CC7, forceps major; CG, cingulum; CST, corticospinal tract; CWM, total cerebral white matter; FA, fractional anisotropy; FX, fornix; HBE, high blast exposure group; IFOF, inferior fronto-occipito fasciculus; ILF, inferior longitudinal fasciculus; L, left; LBE, low blast exposure group; MD, mean diffusivity; smTBI, moderate-severe traumatic brain injury; mTBI, uncomplicated mild traumatic brain injury; R, right; RD, radial diffusivity; SCP, superior cerebellar peduncle; SLF, superior longitudinal fasciculus; UF, uncinate fasciculus.

When comparing the high blast exposure group to the no blast exposure group, none of the findings survived correction for multiple comparisons. Before correction for multiple comparisons, high blast exposure was associated with decreased AD of the bilateral cerebral white matter and uncinate fasciculus, left fornix, and right ATR (βs = −0.238 to −0.187, *p*s < 0.03). It was also associated with decreased RD in the left arcuate fasciculus and CST and decreased MD in the left cerebral white matter, arcuate fasciculus, CST, and fornix (βs = −0.219 to −0.165, *p*s < 0.05). High blast exposure was also associated with increased FA in the left CST, ILF, and SCP (βs = 0.178–0.213, *p*s < 0.05). Again, none of the above findings survived the Benjamini-Hochberg FDR correction. Models were rerun with the low and high blast exposure groups combined into a single any blast exposure group in order to investigate whether increasing power would alter results. Findings were similar to the above analyses, with no relationships between any blast exposure and DTI metrics surviving FDR correction.

After applying the FDR correction, mTBI was a significant predictor of AD in the left (β = −0.200, *p* = 0.003) and right (β = −0.174, *p* = 0.009) SLF-III, left (β = −0.214, *p* = 0.002) and right (β = −0.255, *p* = 0.0001) arcuate fasciculus, and left cingulum (β = −0.243, *p* = 0.0003). We reran these five regressions including only injured controls and removing the non-injured controls. When the non-injured controls were excluded, the relationship between mTBI and white matter integrity was reduced, though mTBI was a significant predictor of AD in the left SLF-III (β = −0.178, *p* = 0.015), left (β = −0.202, *p* = 0.007) and right (β = −0.212, *p* = 0.004) arcuate fasciculus, and left cingulum (β = −0.218, *p* = 0.003), but not right SLF-III AD (β = −0.114, *p* = 0.115), before correction for multiple comparisons. None of these relationships survived FDR correction for 26 comparisons.

### Relationship between post-traumatic stress disorder and diffusion tensor imaging

In order to explore whether a modified DSM-IV diagnosis of PTSD was related to altered white matter integrity, we ran a series of exploratory multi-variate regression models identical to those above, but with the addition of modified PTSD diagnosis. Only one of the 104 models (26 comparisons for each of the four diffusion metrics) demonstrated that PTSD was a significant predictor of white matter integrity before correction for multiple comparisons (R SLF-III RD; β = 0.148, *p* = 0.024), and this did not survive correction for multiple comparisons.

## Discussion

Overall, in this large study investigating self-reported lifetime blast exposure and white matter integrity, we found no evidence to suggest an impact of blast exposure on white matter integrity in service members with and without a history of mTBI. This stands in contrast to past work suggesting decreased FA^14,16^ and increased RD^14^ in a number of different regions as blast exposure increases. Importantly, however, our findings echo two previous smaller studies, one of which found no change in white matter integrity from before to after a breacher training course,^15^ and another in veterans which did not find any relationship between blast exposure and white matter integrity after correction for multiple comparisons.^6^

In contrast to our findings regarding blast exposure, we did find reduced AD (indicating axonal degeneration) in the bilateral superior longitudinal fasciculus and arcuate fasciculus and the left cingulum in SMVs with a history of mTBI compared to those without a history of mTBI. These findings, interestingly, are discrepant with our past work revealing no differences that survived correction for multiple comparisons between persons with a history of mTBI and injured controls.^54^ Because of the focus on blast exposure, the current study included both injured and non-injured controls. In order to determine whether the differences between studies was the result of the inclusion of non-injured controls, we reran these models excluding non-injured controls and found that the associations were reduced, with none of them surviving correction for multiple comparisons. Overall, this suggests that when compared to an injured control group, there are no meaningful differences in white matter integrity a year or more subsequent to uncomplicated mTBI. This is consistent with several studies in the extant literature.^55–58^ These findings also continue to underscore the importance of appropriate control groups when investigating the impact of mTBI. Significant differences are frequently substantially attenuated when healthy control groups are replaced with injured control groups.^9^

Perhaps not surprisingly, and consistent with past research,^24,59–62^ we found a strong relationship between blast exposure and PTSD severity. In contrast, there was no evidence of a relationship between PTSD and white matter integrity, as previously suggested by some,^63,64^ but not all,^6,65,66^ earlier studies. This finding echoed a past finding in an overlapping sample of 116 SMVs with and without mTBI assessed ≥2 years post-injury^67^; however, the present study was conducted in a much larger sample and included non-injured controls as well as injured controls who reported significant symptoms of PTSD but did not have history of TBI. Although some have suggested that blast exposure may result in neurological vulnerability making one more susceptible to PTSD,^6^ our data do not support that any such vulnerability is long-lasting. Instead, it seems most parsimonious that increased lifetime blast exposure serves as a proxy for exposure to traumatic events, with opportunity for the development of PTSD with each event.^32,62^

Limitations of the current study include that it was conducted at least a year after the mTBI or bodily injury. These findings do not speak to acute relationships between mTBI and white matter integrity. Similarly, this study focused only on white matter integrity; other advanced neuroimaging metrics (e.g., resting-state MRI) were not assessed and may have revealed an effect of blast exposure. Other methods of blast exposure assessment, such as consideration of strength of and/or distance from the blast, may have revealed different findings. For instance, Robinson and colleagues found that exposure to a blast within a distance of ≤10 m or blast-related TBI, but not exposure to blasts at greater distances or non-blast-related to TBI, were associated with altered functional connectivity of the default mode network compared to persons without blast exposure within 100 m.^30^ These findings were replicated in a unique sample.^31^ In contrast, exposure to blast(s) within 10 m has been shown to be unrelated to alterations in cerebral blood flow.^68^

Additionally, lifetime blast exposure was assessed with a single interview question. It is likely that differences in reporting style and how one defines blast exposure resulted in some persons with similar blast exposure history being placed in different groups.^32^ There are several measures currently under investigation to improve the assessment of blast exposure history through more in-depth exploration of exposure type and frequency.^28,69,70^ Even such interviews, however, do not guarantee an accurate account of lifetime blast history, which may be confounded by time in general,^71,72^ or other issues that impact reporting style, such as PTSD symptoms^73^ or secondary gain.^74^ Nevertheless, this study is an important addition to the existing literature investigating blast exposure and white matter integrity. The large sample size, use of white matter tractography analysis, and comprehensive TBI history interviews are important strengths of this study.

## Conclusion

Overall, there was no evidence of a relationship between self-reported lifetime blast exposure and white matter integrity in this large study. Additionally, our findings do not support a relationship between remote mTBI history or current PTSD and white matter integrity assessed at least a year after injury. Future longitudinal research with blast exposure metrics is warranted to fully understand any possible impacts of blast exposure on white matter integrity.

## Abbreviations Used

AD: axial diffusivity
ATR: anterior thalamic radiation
CST: corticospinal tract
CT: computed tomography
CWM: total cerebral white matter
DSM-IV: Diagnostic and Statistical Manual of Mental Disorders, Fourth Edition
DSM-IV-TR: Diagnostic and Statistical Manual of Mental Disorders, Fourth Edition, Text Revision
DTI: diffusion tensor imaging
FA: fractional anisotropy
FDR: false discovery rate
GCS: Glasgow Coma Scale
IFOF: inferior fronto-occipito fasciculus
ILF: inferior longitudinal fasciculus
LOC: loss of consciousness
MD: mean diffusivity
MRI: magnetic resonance imaging
mTBI: uncomplicated mild traumatic brain injury
PCL: Post-traumatic Stress Disorder Checklist
PTA: post-traumatic amnesia
PTSD: post-traumatic stress disorder
RD: radial diffusivity
RESTORE: robust estimation of tensors by outlier rejection
SCP: superior cerebellar peduncle
SLF: superior longitudinal fasciculus
SMVs: service members and veterans
T1W: T1-weighted
TBI: traumatic brain injury
TOI: tract-of-interest
